# Kent Campbell, ASTMH Past President, (1944–2024)

**DOI:** 10.4269/ajtmh.24-0627

**Published:** 2024-10-29

**Authors:** Richard W. Steketee, Stephen L. Hoffman

**Affiliations:** ^1^ PATH – MACEPA, Seattle, Washington;; ^2^ Sanaria Inc., Rockville, Maryland

The American Society of Tropical Medicine and Hygiene (ASTMH) mourns the loss of 2007 President Carlos (Kent) Campbell, MD, MPH, FASTMH. Dr. Campbell passed away in Tucson, AZ, on February 20, 2024, at age 80. Obituaries have been published elsewhere,^1^^–^^4^ but we wish to briefly summarize the remarkable impact that our friend Kent had on international public health, the malaria community, the ASTMH, and us.

Known to many for his kind, charismatic demeanor, visionary thinking, and good-natured sense of humor expressed with a southern drawl, Dr. Campbell was a global leader in the fight against malaria, having a profound impact on efforts to control and eliminate the disease. He was an ASTMH Board member from 1991 to 1994 and received the Society’s Joseph Augustin LePrince Medal, which recognizes outstanding work in the field of malariology, in 2012. He delivered his visionary presidential address in 2007: “Africa Tells Us the Story of What Success in Malaria Control Means, Now and in the Future”.^5^

Dr. Campbell was born in East Tennessee and was the namesake of his paternal grandfather, Carlos Campbell, a renowned naturalist and a founder of the Great Smoky Mountains National Park. Kent met Liz (Eliza), the love of his life, in kindergarten, and they married in 1966 to begin a grand adventure together. Over the next fifty-eight years they raised two children and enjoyed four grandchildren.

After completing his pediatric residency at Massachusetts General Hospital and Children’s Hospital in Boston, and an MPH at Harvard School of Public Health, Kent joined the CDC as an Epidemic Intelligence Service Officer in 1972 and was soon deployed to Sierra Leone to investigate a Lassa fever epidemic. A few weeks later he became ill with a possible diagnosis of Lassa fever; this episode was described in “*The Coming Plague*”,^6^ by Laurie Garrett and “*Fever! The Hunt for a New Killer Virus”*,^7^ by John Fuller.

Dr. Campbell’s CDC career in malaria began in the mid-1970s. He and his family spent four years in El Salvador working with the Ministry of Health on nationwide malaria control. Of note, El Salvador became the first Central American nation to be awarded WHO certification of malaria elimination in February 2021.

**Figure:**
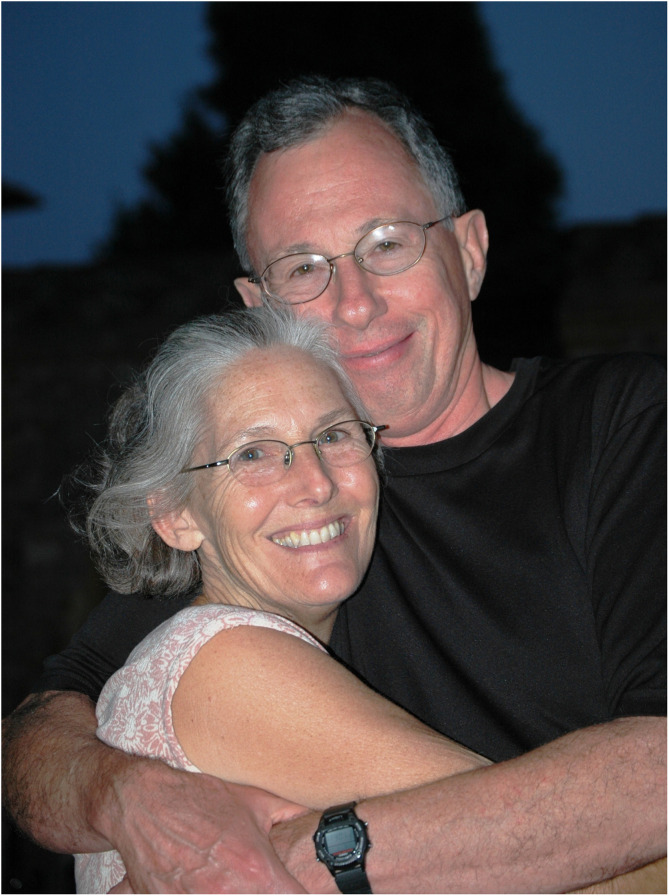
Kent Campbell, ASTMH Past President

Returning to Atlanta, Kent served as Chief of the CDC Malaria Branch from 1981 through 1993, where his team led important efforts to characterize the epidemiolocal and immunological determinants of malaria risk, advance therapies for drug-resistant malaria, evaluate the impacts of malaria, explore prevention strategies for pregnant women and infants, and demonstrate the effectiveness of insecticide-treated mosquito nets (ITNs) to prevent malaria morbidity and mortality. These included the first study showing that when cultured *Plasmodium falciparum* gametocytes were fed to *Anopheles* mosquitoes, they produced sporozoites that could infect a primate. During this period and subsequently, Kent served on numerous expert advisory committees in the U.S. and for the WHO and conferred with many national malaria program managers, especially in sub-Saharan Africa.

In 1995, following his service with CDC, Kent and Liz moved to Tucson where Kent joined the University of Arizona and led the development of the University of Arizona College of Public Health. Following the College’s accreditation, he served as Interim Dean for two years. To give back to the college, Kent and Liz established the Kent & Liz Campbell International Public Health Internship, supporting outstanding Master’s in Public Health students committed to bringing public health to global communities.

After stepping down from the University of Arizona, Dr. Campbell became a consultant to UNICEF on malaria from 2002 to 2004, and in 2003 became a consultant to the Bill & Melinda Gates Foundation, where he contributed to the development of the Foundation’s support for malaria control programs in Africa. This led to his founding of the Malaria Control and Elimination Partnership in Africa (MACEPA) at PATH in 2004. He served as MACEPA’s director until 2008, when he became director of the PATH Malaria Control and Elimination Program and developed and led the PATH Malaria Center of Excellence. Under Dr. Campbell’s leadership, MACEPA partnered with an increasing number of African nations and developed the experience and standards that influenced and supported malaria control programs in over 40 African nations across the continent, where more than 90% of malaria morbidity and mortality occurs.

Notable among MACEPA’s work under Kent’s leadership was its close collaboration and partnership with the Zambian government to implement the Scale-up for Impact (SUFI) program. This effort to rapidly deliver proven malaria tools at scale resulted in Zambia becoming the first country in Africa to successfully achieve high coverage of ITNs, indoor spraying of insecticides, preventive treatment for malaria during pregnancy, and new malaria diagnostics and medications. The SUFI approach soon became the standard for malaria control, informed key elements of the WHO Global Malaria Technical Strategy and was included as a core component of the Global Malaria Action Plan of the Roll Back Malaria Partnership.

As Dr. Campbell noted in 2014, “Building a set of systems changes forever the potential of African communities: that’s what’s kept me in this business. When I started, malaria was merely a biological entity people studied in the lab, and I’ve seen a rapid shift during my career.”

Kent Campbell has left an indelible mark on the ASTMH, the field of global health, all the organizations with which he worked, and the malaria research and control communities. Kent helped launch the malaria and public health careers of his many trainees who he typically treated as family. Kent made the world a better place through his dedication to global public health and there are hundreds of millions whose lives and health were improved through his untiring efforts to control malaria. But Kent was rooted in his dedication to his family; he asked only to be remembered as a loving husband, father, grandfather, and son of the Smoky Mountains.
